# Large tandem chromosome expansions facilitate niche adaptation during persistent infection with drug-resistant *Staphylococcus aureus*

**DOI:** 10.1099/mgen.0.000026

**Published:** 2015-08-03

**Authors:** Wei Gao, Ian R. Monk, Nicholas J. Tobias, Simon L. Gladman, Torsten Seemann, Timothy P. Stinear, Benjamin P. Howden

**Affiliations:** ^1^​ Microbiological Diagnostic Unit Public Health Laboratory, University of Melbourne, Doherty Institute for Infection and Immunity, Parkville, Victoria 3010, Australia; ^2^​ Department of Microbiology and Immunology, University of Melbourne, Doherty Institute for Infection and Immunity, Parkville, Victoria 3010, Australia; ^3^​ Victorian Life Sciences Computation Initiative, University of Melbourne, Parkville, Victoria 3010, Australia; ^4^​ Infectious Diseases Department, Austin Hospital, Heidelberg, Victoria 3084, Australia

**Keywords:** Staphylococcus aureus, antibiotic resistance, chromosome duplication, genomics, mutation, evolution

## Abstract

We used genomics to study the evolution of meticillin-resistant *Staphylococcus aureus* (MRSA) during a complex, protracted clinical infection. Preparing closed MRSA genomes from days 0 and 115 allowed us to precisely reconstruct all genetic changes that occurred. Twenty-three MRSA blood cultures were also obtained during treatment, yielding 44 colony morphotypes that varied in size, haemolysis and antibiotic susceptibility. A subset of 15 isolates was sequenced and shown to harbour a total of 37 sequence polymorphisms. Eighty per cent of all mutations occurred from day 45 onwards, which coincided with the appearance of discrete chromosome expansions, and concluded in the day 115 isolate with a 98 kb tandem DNA duplication. In all heterogeneous vancomycin-intermediate *Staphylococcus aureus* isolates, the chromosomal amplification spanned at least a 20 kb region that notably included *mprF*, a gene involved in resistance to antimicrobial peptides, and *parC*, an essential DNA replication gene with an unusual V463 codon insertion. Restoration of the chromosome after serial passage under non-selective growth was accompanied by increased susceptibility to antimicrobial peptide killing and reduced vancomycin resistance, two signature phenotypes that help explain the clinical persistence of this strain. Elevated expression of the V463 *parC* was deleterious to the cell and reduced colony size, but did not alter ciprofloxacin susceptibility. In this study, we identified large DNA expansions as a clinically relevant mechanism of *S. aureus* resistance and persistence, demonstrating the extent to which bacterial chromosomes remodel in the face of antibiotic and host immune pressures.

## Data Summary

1. Supplementary Table S4 is available through FigShare, http://dx.doi.org/10.6084/m9.figshare.1394626 (2015) Table S4
2. Complete genome sequences available through FigShare pending GenBank release: http://dx.doi.org/10.6084/m9.figshare.1391325 (2015) JKD6229.gb http://dx.doi.org/10.6084/m9.figshare.1391322 (2015) JKD6210.gb http://dx.doi.org/10.6084/m9.figshare.1391323 (2015) JKD6210p.gb
3. Software used available through GitHub https://github.com/tseemann/prokka (2015) and http://www.vicbioinformatics.com/software.shtml
4. Sequence read data for the study isolates have been submitted to the EMBL Sequence Read Archive (ENA). http://www.ebi.ac.uk/ena/data/view/ERP010276

## Impact Statement

Bacterial antibiotic resistance is an increasingly serious global problem. It is well known that resistance gene acquisition or small changes in specific bacterial genes called point mutations can lead to antibiotic resistance. In this study, we reveal yet another means by which the hospital superbug *Staphylococcus aureus* is evading our best antibiotic treatments. We have discovered that during human infection the bacterium makes multiple copies of additional segments of its DNA, increasing the number of genes in specific sections of its chromosome. We show that such gene expansion helps *S. aureus* to beat the last-line antibiotic vancomycin and evade human immune responses. This study reveals yet another tool in the arsenal of *S. aureus* that helps explain why it is such a formidable pathogen and often so difficult to treat.

## Introduction

Nosocomial infections are a major contributor to human morbidity and mortality globally. *Staphylococcus aureus*, a human commensal and well-endowed opportunistic pathogen, is one of the major culprits, especially antibiotic-resistant strains such as meticillin-resistant *S. aureus* (MRSA) (Lowy, 1998). The success of *S. aureus* as a hospital-acquired pathogen is in part due to the capacity of the bacterium to continually evolve and adapt to the hospital environment, frequently leading to enhanced antimicrobial resistance through resistance gene acquisition or point mutations in specific genes (Chambers & Deleo, 2009) and other genetic changes promoting hospital adaptation (Paulander *et al.*, 2013).

The application of new genomics tools to human pathogens has provided insight into the evolutionary dynamics and adaptive mechanisms during colonization or chronic infection that promote persistence and transition from colonizer to invading pathogen (McAdam *et al.*, 2011; Smith *et al.*, 2006; Young *et al.*, 2012). To date, these studies have demonstrated that, contrary to traditional concepts, the bacterial population is heterogeneous during chronic colonization or infection with the accumulation of nucleotide polymorphisms potentially contributing to niche adaptation in the host. During human infection, antibiotic exposure provides a major selection pressure driving such *in vivo* adaptation (Howden *et al.*, 2011; Mwangi *et al.*, 2007), with some antibiotic resistance-associated mutations having been shown to promote resistance to host innate immunity, providing an important link between antibiotic resistance and bacterial persistence (Gao *et al.*, 2013). Despite these recent studies, our understanding of how bacterial pathogens, particularly *S. aureus*, evolve and adapt during persistent infection remains incomplete.

Technological advances have provided the capacity to sequence large numbers of bacterial genomes in a cost-effective, time-efficient manner, providing the potential for new insights into bacterial adaptation during infection. Whilst a growing number of bacterial species are now represented by closed, fully annotated genomes, proportionally most bacterial genome sequences are incomplete (Chain *et al.*, 2009). Comparative approaches based on read-mapping and single nucleotide polymorphism (SNP) or short insertion/deletion (indel) detection are routinely used to analyse and compare incomplete genome sequences (Gao *et al.*, 2010; Howden *et al.*, 2011; McAdam *et al.*, 2011; Mwangi *et al.*, 2007; Young *et al.*, 2012). These approaches detect important changes occurring during infection and have demonstrated the heterogeneous nature of infecting bacterial populations, but they may fail to detect other significant genetic changes, such as the expansion of multicopy insertion sequences (McEvoy *et al.*, 2013), chromosomal duplications (Sandegren & Andersson, 2009) or large genome inversions (Cui *et al.*, 2012). Fully assembled, closed genome sequences and other tools such as optical mapping that generate high-resolution ordered bacterial genome maps provide the means to readily uncover additional chromosomal changes that occur in bacterial species during *in vivo* adaptation.

We have previously sequenced and partially assembled a pair of clinical *S. aureus* from a persistent bloodstream infection with the accumulation of resistance to multiple antimicrobials (Gao *et al.*, 2010). We have shown that point mutations in *rpoB*, *relA* and *rlmN* each contributed to the evolution of antimicrobial resistance and immune escape. However, all the phenotypic changes observed in the final clinical isolate were not fully explained by these three mutations, such as small colony size and vancomycin-intermediate resistance. To better understand the evolutionary dynamics of *S. aureus* during this persistent infection we fully assembled, annotated and recompared the initial (JKD6210) and the final (JKD6229) isolate isolated after 115 days of infection. Additionally, we sequenced 15 intermediate isolates obtained throughout the course of treatment. To the best of our knowledge, this is the first study to show that during human infection, bacterial chromosome expansion in association with sequential acquisition of functionally significant point mutations caused substantial phenotypic changes in the organism, which together promoted antimicrobial resistance and host adaptation.

## Methods

### Isolate details

The details of the clinical case have been reported previously (Gao *et al.*, 2010). The first clinical isolate (JKD6210) was susceptible to vancomycin, rifampicin, ciprofloxacin and linezolid; however, after a complicated treatment course including all four of these antibiotics, a small colony variant (SCV JKD6229) was isolated in the spinal aspirate on day 115 after hospital admission. The SCV JKD6229 was a heterogeneous vancomycin-intermediate *S. aureus* (hVISA) and resistant to rifampicin and ciprofloxacin. It also displayed reduced susceptibility to linezolid. During the 115 days of infection a total of 24 specimens (23 blood cultures and one spinal aspirate) were culture positive for *S. aureus*. In total, 44 different colony morphologies were isolated from the positive cultures. Details of the isolates selected from these cultures are listed in Table S1 (available in the online Supplementary Material). All isolates were grown on horse blood agar (HBA) or in heart infusion broth at 37 °C unless otherwise stated. Colony size was measured on HBA after 20 h incubation.

### δ-Haemolysin assay

The *S. aureus* accessory gene regulator (*agr*) activity was measured by δ-haemolysin production on Trypticase soy agar containing 5 % sheep blood using *S. aureus* strain RN4220, as described previously (Traber *et al.*, 2008). Evidence of enhanced haemolysis between RN4220 and a test isolate was considered positive for δ-haemolysin production. Repression of *agr* was defined as an absence of δ-haemolysin production.

### Antimicrobial susceptibility testing

Isolates were subcultured onto HBA for 18–24 h before performing any antimicrobial susceptibility testing. Vancomycin and teicoplanin macro E-tests were performed by applying 200 μl 2.0 McFarlane bacterial suspension onto brain heart infusion (BHI) agar. The plates were incubated for 48 h before readings were taken. An oxacillin E-test was performed using 0.5 McFarlane suspension onto 2 % NaCl BBL Mueller–Hinton II agar according to the manufacturer's instructions. Agar dilution was used for all other susceptibility testing, according to the Clinical Laboratory Standards Institute. Vancomycin population analysis profile testing was performed on all isolates to detect hVISA, as described previously (Howden *et al.*, 2011).

### Antimicrobial peptide susceptibility testing

Flow cytometry and propidium iodide-stained bacterial cells (excitation, 535 nm; emission, 620 nm; Sigma) were used to determine membrane permeabilization after treatment with antimicrobial peptides (hNP-1 or hBD-2, 20 μg ml^− 1^) as described previously (Gao *et al.*, 2013). Heat-killed bacteria (95 °C for 15 min) and non-peptide-exposed bacteria were used as positive and negative controls, respectively. Flow cytometry was performed (LSRFortessa; Becton Dickinson) in 10 mM K^+^ minimal essential medium, pH 7.2. Fluorescence of a minimum of 5 × 10^3^ cells was acquired for statistical analysis. The analysis was performed in biological triplicates for each peptide/strain combination and data were analysed using FACSDiva software (BD Biosciences).

### Closed genome sequences

The complete genome assemblies for Sa_JKD6210 and Sa_JKD6229 were based on Ion Torrent mate-pair libraries. The libraries were prepared using a demonstration protocol (Ion Torrent p/n 4472004, release date 27 September 2011) with the 5500 SOLiD mate-pair library kit. Libraries were sequenced on an Ion Torrent Personal Genome Machine with a 316D chip and 200 bp chemistry, running TorrentSuite version 3.2.1 (Life Technologies). After processing, 1 629 524 reads for JKD6210 and 2 760 456 reads for JKD6229 contained the mate-pair linker sequence with a mean insert size of 5211 and 5152 bp, respectively. Primary *de novo* assembly was performed using Newbler (version 2.8), resulting in three scaffolds (2 762 210 bp) and two scaffolds (2 727 771 bp), respectively. Genome finishing was managed using Geneious Pro (version 7.2) with gaps closed by PCR, primer walking and Sanger sequencing. Final assemblies were validated by reference to high-resolution *Nco*I chromosome optical maps using MapSolver (version 3.2.0; OpGen). Ion Torrent sequencing errors were corrected by mapping Illumina MiSeq 250 bp paired-end reads from each genome back to the finished sequences (see below). Annotation was performed using Prokka version 1.10 (Seemann, 2014). Chromosome sequences were also compared by blastn analysis and visualized using act (Artemis Comparison Tool; Sanger). Sequences were screened for bacteriophage-associated sequences using phast (Zhou *et al.*, 2011).

### Whole-genome sequencing and analysis

Whole-genome sequencing was performed on the Illumina MiSeq platform, with Nextera XT library preparation and paired-end 250 bp sequencing chemistry. The raw reads were filtered using Trimmomatic (Bolger *et al.*, 2014) to remove ambiguous base calls, Nextera adaptors, bases at Phred quality 10 and reads < 50 bp. Resulting FASTQ sequence read files were read-mapped to the completed JKD6210 genome using Bowtie version 2.1.0 (Langmead & Salzberg, 2012) with default parameters and consensus calling to identify SNPs (indels excluded) using Nesoni version 0.109 (https://github.com/Victorian-Bioinformatics-Consortium/nesoni). A list of the isolates sequenced and summaries of the output for each are shown in Table S1. An alignment file based on the variable nucleotide positions amongst all isolates was used to infer a whole-genome haplotype network using the median-joining algorithm as implemented in SplitsTree version 4.13.1 (Huson & Bryant, 2006).

### Quantitative PCR (qPCR)

Real-time qPCR was used to assay the copy number of selected genes within the chromosomal repeats. The primers are listed in Table S2. Phire Hot Start II DNA polymerase (Thermo Scientific) was used in the qPCR and Evagreen dye (Biotium) was added into reactions in 1 : 20 ratio. The PCR was cycled at 98 °C for 15 s, 52 °C for 18 s and 72 °C for 15 s. The dye signals were sampled during the annealing steps. Standard curves were generated for each pair of primers, using 10-fold serial dilutions of *S. aureus* genomic DNA. The housekeeping gene *gyrB* was used as a reference gene to normalize the results.

### Allelic exchange

By allelic exchange with pKOR1 (Bae & Schneewind, 2006), we introduced the *alr1* 15 bp deletion from BPH1123 into JKD6229. A 2 kb amplicon centred on the deletion was amplified from BPH1123 genomic DNA with primers listed in Table S2 and cloned into pKOR1. The plasmid was electroporated into JKD6229 after isolation from RN4220, with subsequent allelic exchange as described previously (Bae & Schneewind, 2006). Sanger sequencing of the region confirmed the authenticity of the mutation.

### Inducible ParC expression

The full-length *parC* genes from JKD6210 (*parC* WT), SCV JKD6229 (*parC* V463) and BPH1116G20 (*parC* G296D, V463) were amplified by PCR (oligonucleotides used are listed in Table S2). The PCR products were digested with *Kpn*I/*Eco*RI, ligated into plasmid pRAB11 (Helle *et al.*, 2011) and transformed into *Escherichia coli* strain IM08B (Monk *et al.*, 2015). The sequences of the *parC* alleles cloned into pRAB11 were confirmed by Sanger sequencing. The plasmids were directly transformed into JKD6210 with *parC* expression induced by addition of anhydrotetracycline. JKD6210 was also transformed with empty vector as a negative control. The resulting plasmids and strains are listed in Table S3. To assay the impact of inducible *parC* expression, the transformed isolates were grown on Mueller–Hinton agar containing chloramphenicol (10 μg ml^− 1^) with anhydrotetracycline (5 ng ml^− 1^) and incubated for 24 h.

### Repeated subculture of intermediate isolate BPH1116

Isolate BPH1116 was subcultured in BHI broth and passaged daily for 20 days. This involved transfer of 5 μl overnight culture (OD_600_ >2.0) into 10 ml fresh BHI broth. Subculture 20 was named BPH1116-G20. This isolate underwent whole-genome sequencing and qPCR was carried out to assess loss of chromosomal duplications.

## Results

### Isolate characterization during persistent infection

In a previous study, we used genomics to investigate the molecular basis for a *S. aureus* SCV phenotype with reduced linezolid susceptibility, isolated from a single patient over the course of a protracted infection (Gao *et al.*, 2010). Draft genome sequences were obtained for the initial clinical isolate that demonstrated normal colony morphology (JKD6210), and an SCV isolate that had acquired resistance to multiple antimicrobials and was isolated after 115 days of persistent and recurrent infection (JDK6229). Only four mutations (two single nucleotide substitutions, two codon insertions) and the loss of a 25 kb plasmid were found in the SCV. Allelic exchange experiments partially restored the growth defects, but did not fully explain the antimicrobial resistance of the SCV. To better understand the genetic changes that led to the full SCV phenotype, we conducted a more detailed comparative and functional genomic investigation that began by closing and annotating JKD6210 (initial isolate) and JKD6229 (final isolate). We then sequenced 15 of the 44 *S. aureus* isolates that were cultured from 24 clinical samples (23 blood cultures) that had been submitted to the laboratory during the patient's treatment. The *S. aureus* isolates from these samples exhibited extensive phenotypic heterogeneity, especially after day 14 of infection, initially observed as a range of colony morphotypes. For each blood culture plate, a representative of each distinct colony morphotype was stored at − 80 °C for later analysis.

**Fig. 1.**
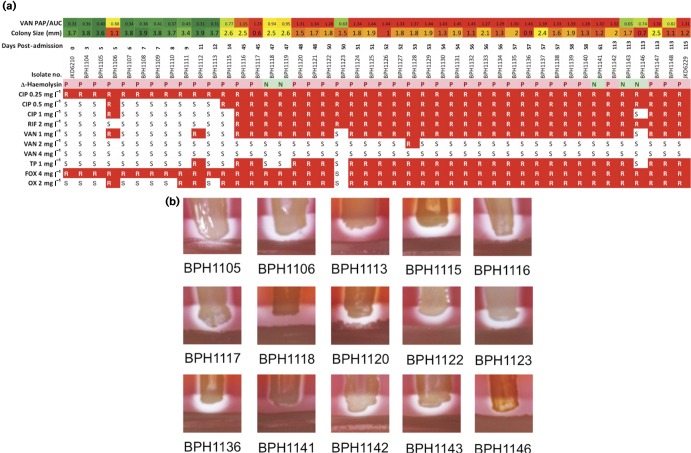
Phenotypic diversity of isolates during the persistent infection. (a) The colony size, antibiogram, vancomycin population analysis profile area under curve (VAN PAP/AUC) result and δ toxin activity of all 44 isolates are summarized. Increasing vancomycin resistance is indicated by colour (green is susceptible and red is resistant). Likewise, reduced colony size is indicated by red, whilst normal colony size is indicated by green. The sequential increase in antibiotic resistance is highlighted in red, with R indicating no growth and S indicating growth on the relevant antibiotic concentration. CIP, ciprofloxacin; RIF, rifampicin; VAN, vancomycin; TP, teicoplanin; FOX, cefoxitin; OX, oxicillin. (b) Analysis of δ-haemolysin production in 15, sequenced intermediate isolates demonstrated significant heterogeneity. Clinical isolate BPH1118 had no δ-haemolysin activity, whilst BPH1141 and BPH1141 had weak activity.

To determine the phenotypic heterogeneity of the different colony types that emerged during the infection, each morphotype was assayed for growth characteristics, haemolysis patterns and antibiotic susceptibility profiles (Fig. 1). This testing included 44 isolates from the 24 clinical samples. The colony diameter sizes were highly variable over the course of the infection (0.6–3.9 mm) and whilst the initial clinical isolate was homogeneous with normal colony morphology, the subsequent cultures demonstrated mixed colony morphotypes (Fig. 1a). Colonies of reduced size were first isolated from the day 5 blood culture. Most blood cultures after day 14 produced mixed populations with varying colony sizes ranging from 0.6 to 2.5 mm (Fig. 1a).

Antibiogram and vancomycin population analysis profile testing demonstrated the sequential acquisition of low-level vancomycin resistance in the bacterial population, although some vancomycin-susceptible populations (BPH1123, BPH1143, BPH1146 and BPH1148) were still present after day 14 where the hVISA strains were dominant in the infection (Fig. 1a). The δ-haemolysin assay is a surrogate marker for activity of the accessory gene regulator (*agr*) locus in *S. aureus*, the major quorum-sensing regulator of virulence. This assay suggested that strains BPH1118 (day 47), BPH1141 (day 61) and BPH1146 (day 113) had defective *agr* function, whilst other strains had intact *agr* function (Fig. 1a).

These phenotypic comparisons clearly indicated that a heterogeneous population of *S. aureus* infected the patient. To better understand the evolution of the bacterial population during this persistent infection, additional genome sequencing was performed. This included the full assembly and closing the genomes for the first and last clinical isolates, and generating draft genome sequences for the 15 intermediary isolates (Table S1).

### Comparison of fully assembled chromosomes reveals a large chromosomal duplication

**Fig. 2.**
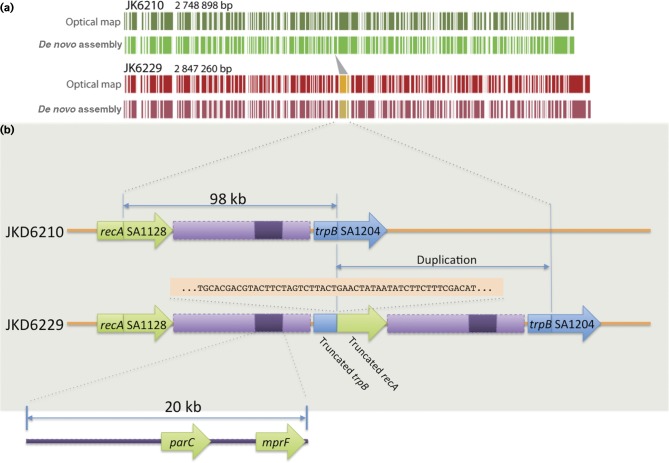
Complete and correct assembly of JKD6210 and JKD6229. (a) Comparison of high-resolution *Nco*I optical maps with *in silicoNco*I predictions confirming correct assembly of the chromosome for each isolate. Depicted also is the 98 kb duplicated region. (b) Expanded view of the 98 kb duplication showing the flanking protein-coding DNA sequence impacted by the mutation, and the location of *parC* and *mprF* within a 20 kb internal segment. Shown also is 50 bp of sequence either side of the *trp*B/*recA* junction.

To enable a thorough analysis of the 15 additional draft genomes sequences, we fully assembled the chromosome for the initial and the final clinical isolates, JKD6210 (single circular chromosome 2 748 898 bp, circular plasmid 25 003 bp) and JKD6229 (single circular chromosome 2 847 260 bp). We utilized a combination of Ion Torrent mate-pair and Illumina paired-end libraries to produce high-quality closed genomes. High-resolution *Nco*I optical maps were additionally generated for both isolates to guide and validate *in silico* scaffolding. Unexpectedly, the optical maps suggested an ∼100 kbp repeated region in the SCV JKD6229 chromosome (Fig. 2a). The *in silico* assembly of JKD6229 confirmed the optical mapping and demonstrated a 98 231 bp sequence in a tandem duplication from *recA* nucleotide position 1 356 561 to *trpB* position 1 454 925 (JKD6210 numbering). The duplication created a hybrid *trpB*/*recA* gene (Fig. 2). The impact of the duplication on phenotype is assessed in a later section.

**Fig. 3.**
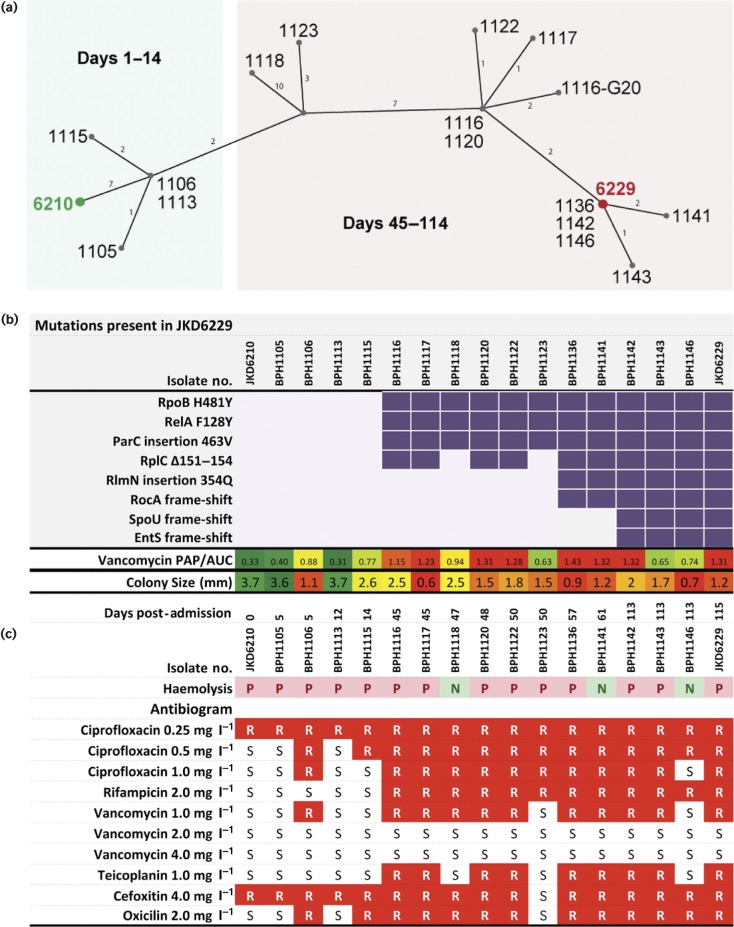
Population genotype and phenotype summary of all sequenced isolates. (a) Median-joining network graph showing the genetic relationship between the 17 clinical *S. aureus* isolates obtained over the duration of treatment, inferred from whole-genome sequence alignments against JKD6210.Numbers on graph edges indicate SNPs. (b) Mutation summary, culminating in SCV JKD6229 as shown by purple blocks. Lower row contains a heatmap summary of changing vancomycin susceptibility. (c) Subset of the phenotype data for each isolate presented in Fig. 1 for ease of comparison with the corresponding genotype, including heatmap of mean colony size (mm) fluctuations, haemolysis and antibiogram results generated by agar dilution. Red blocks denote resistance.

Analysis of the two closed chromosomes also uncovered previously unrecognized mutations in SCV JKD6229, including three small codon deletions in *rplC*, *spoU* and *entS*, and one in-frame insertion in *rocA* (Fig. 3, Table S4).

### Genome sequence comparisons of the intermediate isolates confirm a heterogeneous population

Fifteen intermediate isolates were selected for whole-genome sequencing. Sequence reads were mapped to the JKD6210 completed chromosome and a full list of variants was produced (Figs 3 and S1, Table S4). A haplotype network was inferred using the median-joining algorithm from alignment of the resulting variable nucleotides (indels excluded). This network demonstrated the heterogeneous structure of the *S. aureus* population within the patient, as it evolved from the single progenitor strain (Fig. 3a). The majority of mutations arose after the exposure to rifampicin, ciprofloxacin and vancomycin from day 14 onwards, and this was also reflected in dramatically diverse colony morphotypes. There were eight persisting mutations that were absent in JKD6210 and accumulated in one or more intermediate strains to persist through to JKD6229 (Fig. 3). The critical time period for the emergence of the persistent SCV was between days 14 and 45, when the mutations in *rpoB*, *relA*, *parC* and *rplC* first appeared in BPH1116. This was the first hVISA strain isolated during the infection (Fig. 3a). All four of these mutations were sustained in isolates recovered during the infection except for the absence of the *rplC* mutation in two vancomycin-sensitive *S. aureus* (VSSA) strains (Fig. 3a, b, Table S3). The mutations in *rlmN* and *rocA* were detected at day 57 after linezolid was first employed, with the *rlmN* mutation shown to inactivate RlmN methylase activity and directly associate with linezolid resistance (Gao *et al.*, 2010; LaMarre *et al.*, 2011). Mutations in *spoU* and *entS* appeared in isolates from the day 113 blood culture.

An analysis of the mutations that appeared in one or more of the intermediate isolates but did not persist through to JKD6229 was also undertaken (Fig. S1, Table S4). Some of these changes helped explain the varying phenotypes of the intermediate isolates. For example, BPH1118 had five additional changes that included an *agrA* loss-of-function mutation that explained the absence of δ-haemolysis in this isolate (Fig. 1a). A 15 bp deletion in *alr1* was found in the VSSA isolate BPH1123,. This gene encodes an alanine racemase. Transfer of the *alr1* allele from BPH1123 into the hVISA JKD6229 increased the susceptibility of the strain to vancomycin, which fits with the sensitivity of BPH1123 to vancomycin and oxacillin (Fig. S2).

### Large chromosomal duplications contribute to antibiotic and antimicrobial peptide resistance

**Fig. 4.**
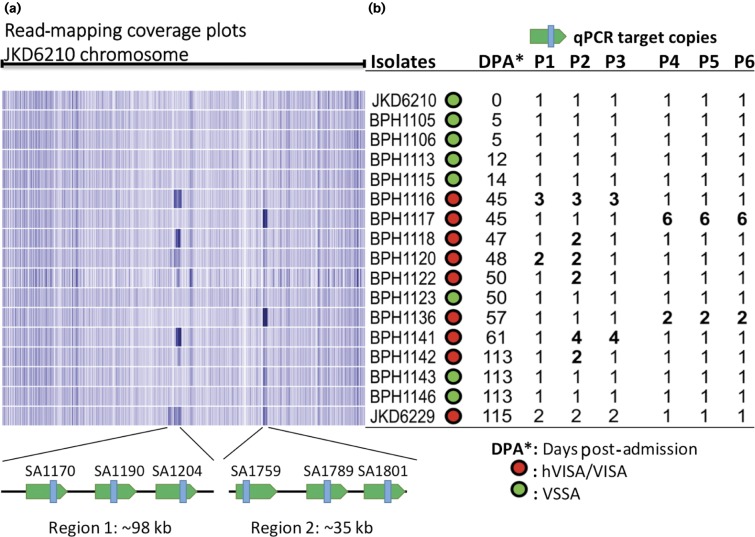
Detection of chromosomal expansions in intermediate isolates. (a) Heatmap sequence coverage summary of the 15 isolates against the chromosome of JKD6210, showing a region of likely chromosomal amplification of variable length from 10 to 98 kb (region 1) present in six isolates and a ∼35 kb region (region 2), corresponding to phiSA3. (b) Three coding sequences from region 1 and 2 were selected as qPCR targets for verifying the copy number of the amplified regions shown in (a). The copy numbers of these coding sequences were aligned with coverage summary and confirm the expected copy number changes. Isolates that demonstrated reduced vancomycin susceptibility are highlighted in red, whilst green indicates vancomycin susceptibility. All isolates containing chromosomal expansions had reduced vancomycin susceptibility. P1–P3, region 1; P4–P6, region 2 (phiSA3).

Given the discovery of the chromosomal duplication in JKD6229, we examined changing the read-coverage density across the JKD6210 chromosome to investigate possible chromosomal duplications amongst the intervening isolates (Fig. 4). Two distinct high-coverage regions suggestive of chromosomal repeats were noted, first detected in BPH1116 at day 45 (Fig. 4a). Region 1 was variable in length, up to ∼98 kb and spanned the genes SA1128–SA1204 (*S. aureus* N315 locus_tag nomenclature). This was the same region duplicated in JKD6229 (Fig. 2). Region 1 also appeared to be duplicated in five other isolates (Fig. 4). We used the unique sequence in JKD6229 across the *trp*B/*recA* junction (Fig. 2b) to screen for the same duplication site within the sequence reads of the intermediary isolates. Only JKD6229 contained this sequence, indicating that this region was independently duplicated amongst the intermediary isolates.

Region 2 was ∼35 kbp and spanned the staphylococcal bacteriophage phiSA3. Higher read-coverage across this region was suggestive of mixed cell populations with integrated and excised circular phage forms, as the strains BPH1117 and BPH1136 were still producing α-haemolysin, which is inhibited if β-haemolysin is made (Fig. 1a). The β-haemolysin gene is disrupted on phiSA3 integration. We therefore conducted an *in silico* screen of the reads from all isolates for the presence of the sequence across the junction of the circular form of phiSA3. Reads from *S. aureus* isolate BPH1116 onwards indicated the presence of excised, circular forms of phiSA3. This result explains the higher read-coverage across region 2. We further confirmed the read-coverage results by qPCR targeting three protein-coding DNA sequences within each of region 1 and region 2 (containing phiSA3) (Fig. 4a). Read-coverage and mapping locations across region 1 indicated there were duplications of variable length (20–98 kb) and copy number (Fig. 4a).

Intriguingly, the read-coverage and confirmatory qPCR showed that only isolates with reduced vancomycin susceptibility contained chromosome expansions, suggesting a possible contribution of this DNA amplification to antibiotic resistance. Isolate BPH1122 contained the shortest example of the region 1 chromosome duplication, covering 11 coding sequences from SA1183 to SA1193 (Fig. 4). These coding sequences included genes encoding proteins involved in DNA replication, in particular chromosome segregation (*parC*, *parE*), and *mprF*, encoding multiple peptide resistance factor (MprF). In *S. aureus*, mutations leading to increased activity of MprF are associated with enhanced resistance to host defence peptides and reduced susceptibility to daptomycin (Bayer *et al.*, 2014). Only one mutation was detected across this region compared with JKD6210 and this was the V463 codon insertion that was present in all copies of *parC*, first observed in the day 45 isolate BPH1116 (Figs 3a and 4). Mutations in *parC* are associated with fluoroquinolone resistance, but usually occur at or around position 80 (Schmitz *et al.*, 1998).

**Fig. 5.**
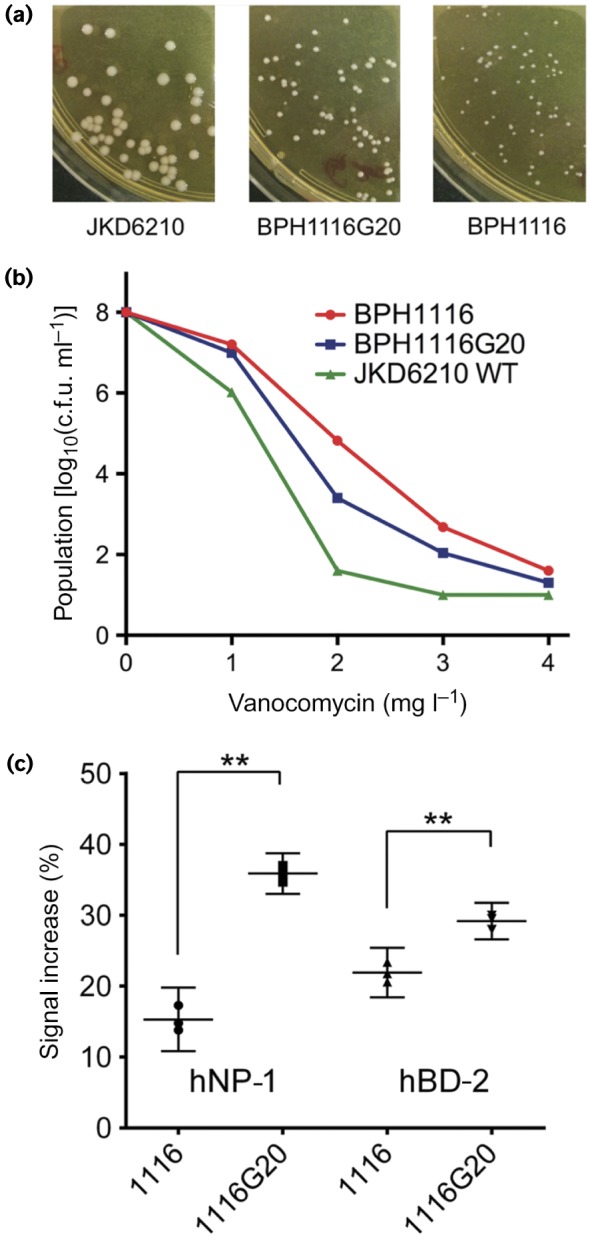
Impact of chromosomal expansion on key phenotypes. (a) Comparisons of colony morphology, showing partial restoration of colony size after loss of the chromosomal amplification spanning *parC* and *mprF* in isolate BPH1116G20.(b) Increased susceptibility to vancomycin upon loss of the chromosomal amplification as measured by population analysis profile. (c) Increased sensitivity to antimicrobial peptide killing upon loss of the chromosomal amplification as indicated by enhanced propidium iodide staining, as measured by flow cytometry. The *y*-axis shows the increase in propidium iodide-mediated fluorescence for antimicrobial peptide-exposed versus non-exposed *S. aureus.* Results indicate means and 95 % confidence intervals for biological triplicates. **Significantly different, *P* < 0.001, unpaired *t*-test.

To investigate the functional impact of the region 1 chromosomal duplication in *S. aureus*, isolate BPH1116 (3 × 60 kb tandem repeated sections) was serially subcultured for 20 days, generating BPH1116G20 which was shown by qPCR and genome sequencing to have lost the chromosomal expansion (Fig. S3). When BPH1116 (day 45) was compared with JKD6210 (day 0), it exhibited a reduced colony size and susceptibility to vancomycin (Fig. 1a), with 3 × 60 kb copies of the region 1 chromosomal duplication present (Fig. 4). BPH1116G20 was tested for the above phenotypes and additionally screened for the resistance to host defence peptides, due to the presence of *mprF* in the amplified region. BPH1116G20 displayed increased colony size, susceptibility to vancomycin and host defence peptides compared with BPH1116 (Fig. 5).

In addition to the loss of the chromosomal duplication, genome sequencing of BPH1116G20 identified two additional non-synonymous point mutations, one each in the genes *parC* and *abgT* (AbgT^G326R^). The gene *abgT* encodes a putative *p*-aminobenzoyl-glutamate transport protein, which may be involved in uptake of folate degradation products (Green *et al.*, 2010). The additional mutation in *parC* that yielded ParC^G296D/V463^ (compared with JKD6210) was particularly interesting given the essential nature of *parC* and that three copies of ParC^V463^ were present in the chromosomal duplication within BPH1116 (Fig. 4). Despite numerous attempts, we were unable to introduce the V463 allele into the WT *parC* of JKD6210.

### Deleterious effect of overexpression of *parC* V463 in *S. aureus*

**Fig. 6.**
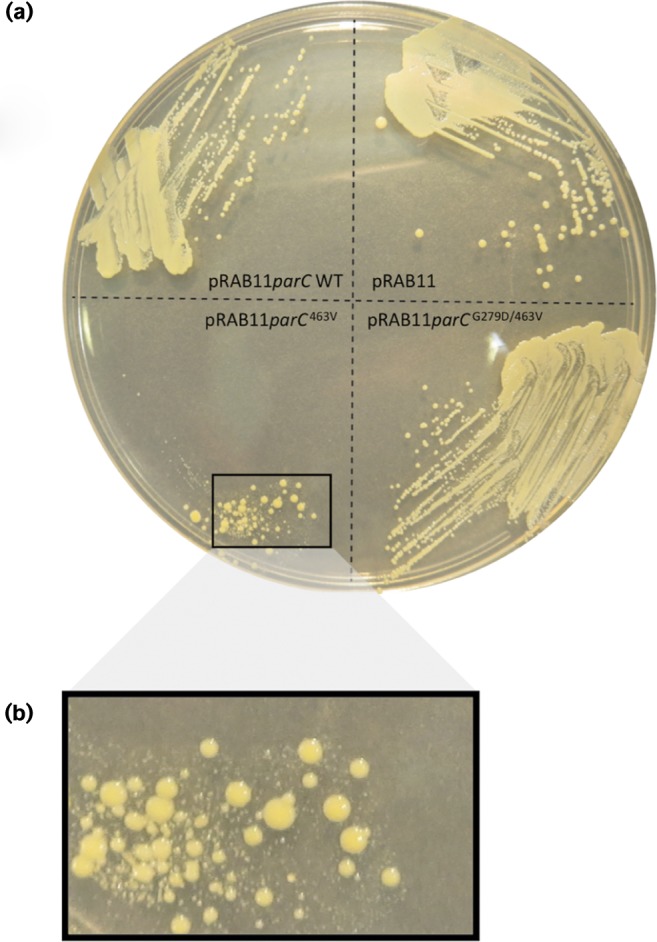
Impact of ParC mutations on growth of *S. aureus* JKD6210.(a) Expression of three different *parC* alleles in JKD6210. The alleles were cloned into the Tet-inducible expression vector pRAB11 and transformed into JKD6210. Shown is growth of each strain on Mueller–Hinton agar in the presence of 5 ng anhydrotetracycline ml^− 1^.(b) Impact of overexpression of *parC* with the V463 mutation on growth and colony size of JKD6210.

We therefore took an alternate approach and placed the three different *parC* alleles [WT, V463 and the double mutation (V463, G296D)] under a Tet-inducible promoter in the plasmid pRAB11 and expressed them in the JKD6210 background. Using only a very low level of inducer (concentrations of >80 ng anhydrotetracycline ml^− 1^ were toxic for all alleles, including WT), the growth characteristics of the two *parC* mutants were compared with controls (Fig. 6). Expression of *parC* V463 had a profound impact on growth; a phenotype that was rescued in the presence of the G296D mutation suggesting that this mutation is compensatory (Fig. 6). We also assessed the susceptibility of each strain to ciprofloxacin by E-test ( < 5 ng anhydrotetracycline ml^− 1^), but observed no change in resistance for either of the mutants compared with WT (data not shown). We also wondered if the ParC^V463^ allele induced a mutator phenotype in *S. aureus*. To test this, we compared the frequency of occurrence of spontaneous rifampicin resistance in each of the strains < 5 ng anhydrotetracycline ml^− 1^ induction, but we observed no difference in mutation frequency between strains harbouring mutants and WT *parC* alleles (observed range 2.1 × 10^− 8^ to 3.6 × 10^− 8^).

## Discussion

Recent advances in genomics have provided the tools to precisely unravel the genomic population structure during bacterial infection. However, utilizing these technologies to understand pathogen populations and evolution during clinical infections requires carefully collected and curated clinical isolates. Most studies to date investigating the evolution of *S. aureus* during clinical infections, in particular in relation to evolving antibiotic resistance, have focused on horizontal gene transfer events (Berglund & Söderquist, 2008; Bloemendaal *et al.*, 2010; Weigel *et al.*, 2003), or the sequential acquisition of SNPs or small indels (Cameron *et al.*, 2012; Friedman *et al.*, 2006; Howden *et al.*, 2008, 2011; Mwangi *et al.*, 2007; Weigel *et al.*, 2003), usually by comparing consensus sequences from a pair of clinical isolates with divergent susceptibility profiles. In this study, by carefully characterizing phenotypic diversity within the infecting *S. aureus* population recovered from a serious and persistent *S. aureus* infection, we identified a continuously evolving bacterial population within the patient that facilitated adaptation to higher levels of antibiotic resistance, reduced susceptibility to host innate immune factors and altered virulence. The phenotypic and genomic diversity revealed within a single patient infection highlights the population complexity of high-burden bacterial infections, and also demonstrates that selecting single colonies for sequencing from such cases will not reveal this genomic diversity.

Here, we also showed that high-quality closed genome sequences are important for gaining a deeper understanding of how *S. aureus* is evolving during infection. The large chromosomal duplications we observed were readily detected by comparisons of completed genomes, but were easily missed by the more common automated *de novo* assembly or read-mapping approaches.

The acquisition of mutations during the infection was rapid in an evolutionary sense, driven by the highly selective nature of the environment, including host defence mechanisms and multiple antimicrobial exposures. Whilst eight mutations were sequentially acquired and maintained in the final clinical isolate (Fig. 3), a number of which have been demonstrated to confer survival advantages through activation of the stringent response (RelA fig128Y), reduced host innate immune susceptibility (RelA fig128Y, RpoB H481Y) or enhanced antimicrobial resistance (RpoB H481Y, RlmN 345E insertion) (Gao *et al.*, 2010, 2013), 39 SNPs or indels appeared in an apparently transient manner and were not selectively maintained. Investigation of one transient 12 bp deletion in *alr1*, encoding alanine racemase 1 (which converts l-alanine to d-alanine, a cell wall precursor), found in isolate BPH1123, restored vancomycin susceptibility in the day 50 isolate (Fig. S2). The mutation resulted in increased antibiotic susceptibility, possibly explaining its lack of persistence. The additional mutations maintained in strain JKD6229 are also predicted to contribute to the altered growth and reduced antibiotic susceptibility of this isolate. The 5 bp frame-shift mutation in *rocA* is predicted to result in a dysfunctional oxidoreductase and impaired l-proline degradation in the arginine deaminase pathway. This may result in a lower efficiency of the arginine deaminase pathway to provide ATP. The downregulation of *rocA* was previously reported in a *hemB*-mutated SCV (Seggewiss *et al.*, 2006), suggesting that this insertion may contribute to the SCV phenotype. The 12 bp deletion in *rplC* results in a 4 aa deletion in ribosomal protein L3, a mutation that may contribute to the slow growth of the isolate (Cui *et al.*, 2010) and the reduced linezolid susceptibility (Locke *et al.*, 2009). The impacts of the frame-shift mutations in *spoU*, encoding the TrmH family RNA methyltransferase, and *entS*, encoding a putative major facilitator transporter, are unclear (Table S4).

Aside from SNPs and indels, distinct chromosomal rearrangements and duplications have received increasing attention as mediators of bacterial evolution and adaptation (Sandegren & Andersson, 2009; Sun *et al.*, 2012), especially as mediators of enhanced antimicrobial resistance (Sandegren & Andersson, 2009). These gene duplications and amplifications generate extensive and reversible genetic variation that facilitates bacterial adaptation (Andersson & Hughes, 2009). The most striking feature of the genomic analysis of isolates from this infection was the variable chromosomal duplications that were uncovered only in isolates demonstrating an hVISA phenotype. Gene duplications and amplifications have been studied predominantly *in vitro*, reported rarely clinically and, to the best of our knowledge, have never been reported during human infection with *S. aureus.* Interestingly, the size, copy number and precise break-points of the duplicated regions were variable amongst the intermediate isolates, with the smallest detected region containing only 11 coding sequences, whilst the largest duplication was ∼98 kb. These data indicate that chromosome duplications were occurring independently in this region over the course of the infection. Several mechanisms have been proposed to explain the occurrence of gene duplications and amplifications (Sandegren & Andersson, 2009). Directly oriented repeated sequences such as insertion sequence elements or rRNA operons can mediate a RecA-dependent non-equal homologous recombination. However, the absence of repeated sequences flanking the duplicated region here suggests a RecA-independent mechanism, which may include phenomena such as DNA break-mediated rolling circle replication (Sandegren & Andersson, 2009). Once a region has been duplicated then RecA-dependent mechanisms may be involved in producing additional copies, using the tandem duplication as substrate for homologous recombination. Such a process could explain the presence of 3 × 60 kb copies of the region observed in BPH1116 (Fig. 4).

Chromosomal duplications confer a fitness cost on bacteria (Adler *et al.*, 2014), hence they tend to be unstable. Serial passage of isolate BPH1116 resulted in loss of the chromosomal duplication, and comparison of this isolate (BPH1116G20) with the isolate containing the duplication revealed important phenotypic consequences of the duplication that included enhanced antibiotic resistance and reduced susceptibility to human antimicrobial peptides. The smallest duplicated region (11 coding sequences) included *mprF*, which has been linked previously to changes in vancomycin susceptibility (Ruzin *et al.*, 2003), and host antimicrobial peptide and daptomycin resistance by altering the surface charge of the cell surface (Ernst & Peschel, 2011; Mishra *et al.*, 2009). Resistance to two human antimicrobial peptides, hNP-1 and hBD-2, was therefore analysed in the intermediate isolate BPH1116 containing multiple copies of *mprF* and the lab-derived BPH1116G20, which only has one copy. As predicted, BPH1116 was more resistant to killing by antimicrobial peptides than BPH1116G20.

We were particularly interested in the role of the unusual V463 codon insertion mutation in the duplicated copies of *parC* (also called *grlA*). Quinolone resistance in *S. aureus* is most frequently caused by substitution mutations at codon 80, within the quinolone resistance-determining region (Schmitz *et al.*, 1998). Mutations at this position confer high-level quinolone resistance (typically >32 μg ml^− 1^). The V463 mutation appears uncommon and, to the best of our knowledge, has not been, reported previously. We expected this mutation might explain the shift observed in susceptibility to ciprofloxacin at day 45 from 0.5 to 1.0 mg l^− 1^ (Fig. 1). Inducible expression of the V463 *parC* allele in JKD6210 saw a substantial impact on colony size and growth, but there was no change in antibiotic susceptibility (Fig. 6). In other bacteria, quinolone resistance mutations can dramatically alter the minimal selective concentration (MSC), sometimes at antibiotic concentrations 230-fold less than the MIC (Gullberg *et al.*, 2011). The MSC is the concentration of antibiotic where the fitness cost of the mutation is balanced by the selective effect of the drug (Sandegren & Andersson, 2009) and we speculate that the two copies of the V463 mutation might be lowering the MSC, giving the strain a competitive advantage in the patient, in circumstances where the concentration of antibiotic (in this case ciprofloxacin) was below the MIC. Experiments to test this hypothesis remain to be performed. Quinolones have also been reported to mediate disturbances in DNA fork progression and replication repair, and we speculate that the ciprofloxacin exposure might have had such an effect (Didier *et al.*, 2011).

This clinical case represents a remarkably resistant and persistent clinical infection. Our genomic deconstruction of the infection provides a window into the complex bacterial dynamics of deep-seated staphylococcal infections, demonstrating for the first time that multi-copy chromosome duplications and amplifications, in addition to the sequential acquisition of diverse mutations, lead to drug-resistant, clinically persistent *S. aureus* isolates.

## Supplementary Data

1047 Supplementary Data
