# Supplementary material for: Large tandem chromosome expansions facilitate niche adaptation during persistent infection with drug-resistant Staphylococcus aureus
Source: Microb Genom. 2015 Aug 3;1(2):e000026. doi: 10.1099/mgen.0.000026 (PMC5320569; doi:10.1099/mgen.0.000026)
Supplement: Supplementary file 1 — Supplementary Data [file mgen-01-26-s001.pdf]

Table S1. Staphylococcus aureus strain and sequence data.

Strain ID number	Day of Infection	Source	Seq platform	Read type	Read length	No. of read pairs	Seq Depth
Sa_JKD6210	0	Blood culture		closed reference			
Sa_BPH1105	5	Blood culture	Illumina MiSeq	paired	250bp	619749	54.627
Sa_BPH1106	5	Blood culture	Illumina MiSeq	paired	250bp	688303	60.978
Sa_BPH1113	12	Blood culture	Illumina MiSeq	paired	250bp	560463	49.464
Sa_BPH1115	14	Blood culture	Illumina MiSeq	paired	250bp	637561	60.269
Sa_BPH1116	45	Blood culture	Illumina MiSeq	paired	250bp	640443	57.691
Sa_BPH116G20		Laboratory subculture	Illumina MiSeq	paired	250bp	651688	105.067
Sa_BPH1117	45	Blood culture	Illumina MiSeq	paired	250bp	1189751	120.769
Sa_BPH1118	47	Blood culture	Illumina MiSeq	paired	250bp	1182627	119.927
Sa_BPH1120	48	Blood culture	Illumina MiSeq	paired	250bp	1477806	150.865
Sa_BPH1122	50	Blood culture	Illumina MiSeq	paired	250bp	611571	73.731
Sa_BPH1123	50	Blood culture	Illumina MiSeq	paired	250bp	608334	49.627
Sa_BPH1136	57	Blood culture	Illumina MiSeq	paired	250bp	543076	43.987
Sa_BPH1141	61	Blood culture	Illumina MiSeq	paired	250bp	1342475	137.512
Sa_BPH1142	113	Blood culture	Illumina MiSeq	paired	250bp	1320196	135.578
Sa_BPH1143	113	Blood culture	Illumina MiSeq	paired	250bp	1281257	130.317
Sa_BPH1146	113	Blood culture	Illumina MiSeq	paired	250bp	854649	67.843
Sa_JKD6229	115	Spinal Aspirate		closed reference			

Table S2. Oligonucleotides used in this study

Oligo ID	Oligo sequence 5' – 3'	Target	Function
P0092	ACAGCAGGGCCTAGGGGACC	SA1170	qPCR
P0093	TGGCATGCATTCGACGTTCTGGT		
P0100	TCGCCATTCCAAGCATTTGCGAT	SA1190	qPCR
P0101	ACACTGCACCTGGACCGCCT		
P0094	TGAAGCCGCAGGTCAAGGCG	SA1204	qPCR
P0095	TGCTAGTTGCACTTGCCCACCT		
P0096	GCGAAAATCGACGGCTTAGGTGC	SA1759	qPCR
P0097	GGCGCGTATACAGCCTGTCC		
P0098	TAATGCGCCGATGGGGTCGC	SA1789	qPCR
P0099	TGCTTTGTGTAAGACGTTGGCATGT		
P0102	ACATCGGGCAACGCAGATTGT	SA1801	qPCR
P0103	TGTGTGACCGTCCGAATGTGTGA		
P0088	GGGGACAAGTTTGTACAAAAAAGCAGGCTTGGTGAGCTACTGCATTCAGCCCTAA	*alr1*	Allelic exchange
P0089	GGGGACCACTTTGTACAAGAAAGCTGGGTGCAGCCAAAATTGGTTGAGCGGA	*alr1*	
P0072	ACGCGACCAATAACTTCGCATTGA	*alr1*	Confirm mutation
P0073	TGTCAGAACTCAGCAGGCTCTCT	*alr1*	
P0135	TGGATCCCCTCGAGTTCATG	*parC*	Confirm *parC* insertion in pRAB11
P0136	TGTAAAACGACGGCCAGTGA		
P0133	ATATGGTACCGAGGAGGAATTGGAAGTGAGTGAAATAATTCAAGATTTATCACTTG	*parC*	Cloning gene in pRAB11 with incorporation of rbs
P0134	ATATGAATTCTTAGCTAATATACATGTCTATTACTTCACC		

Table S3. Non-clinical *S. aureus* isolates used in this study

Isolates ID	Description
BPH1116G20	The 20th passage of BPH1116 in brain heart infusion broth
BPH1201	*S. aureus* JKD6229 with *alr1* mutation from *S. aureus* BPH1123
BPH1221	*E. coli* IM08B with pRAB11::$parC_{WT}$
BPH1222	*E. coli* IM08B with pRAB11::$parC_{v480}$
BPH1223	*E. coli* IM08B with pRAB11::$parC_{v480,G296D}$
BPH1224	*S. aureus* JKD6210 with pRAB11::$parC_{WT}$
BPH1225	*S. aureus* JKD6210 with pRAB11::$parC_{WT}$
BPH1226	*S. aureus* JKD6210 with pRAB11::$parC_{v480}$
BPH1227	*S. aureus* JKD6210 with pRAB11::$parC_{v480,G296D}$

Figure S1

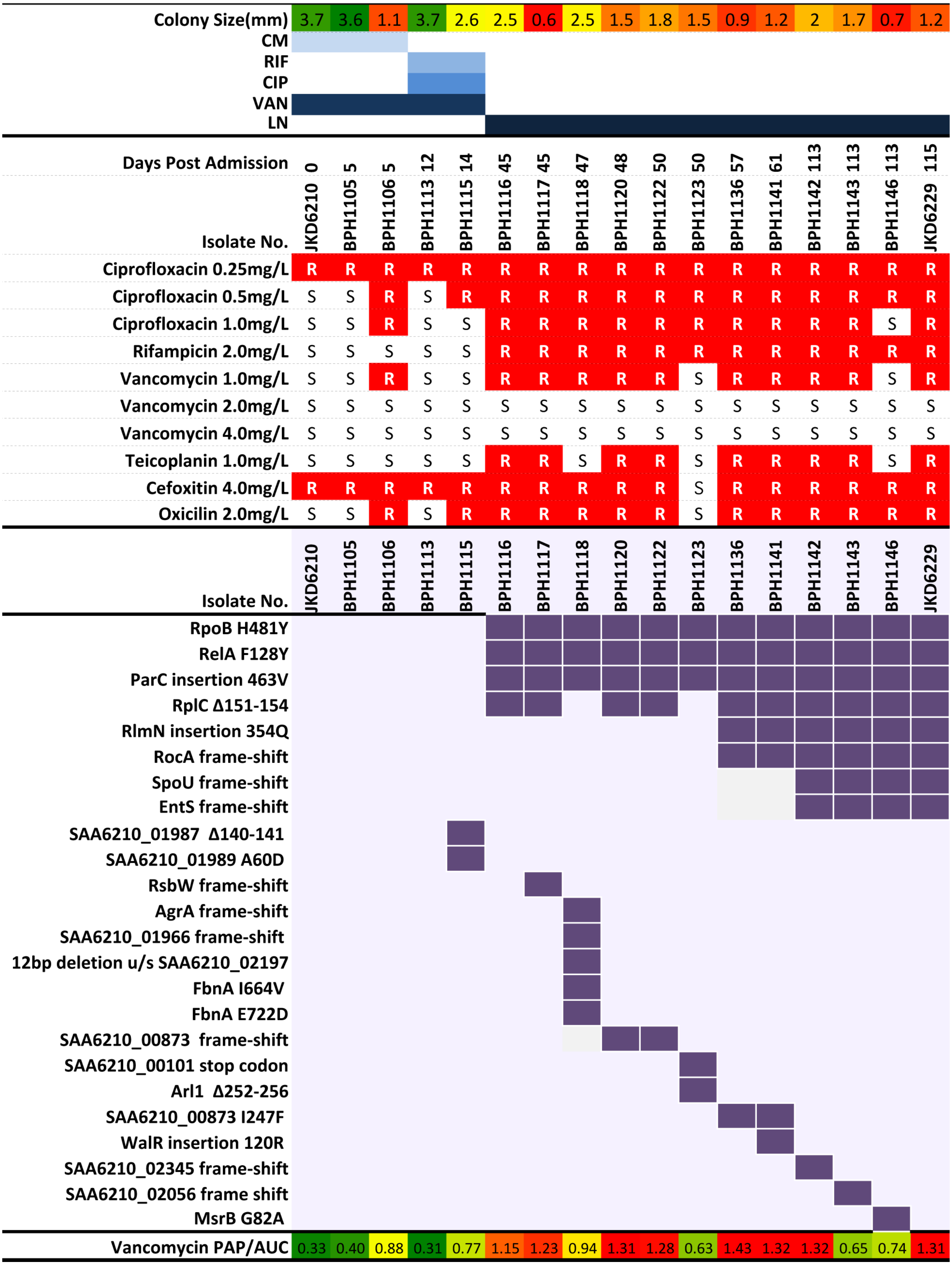

Fig. S1: Phenotype and genotype diversity of isolates during persistent infection. Complete list of functional SNPs and indels occurring across the 115 days of treatment among the 17 S. aureus isolates. Locus_tag positions refer to the genome annotation of Sa_JKD6210

Figure S2

A

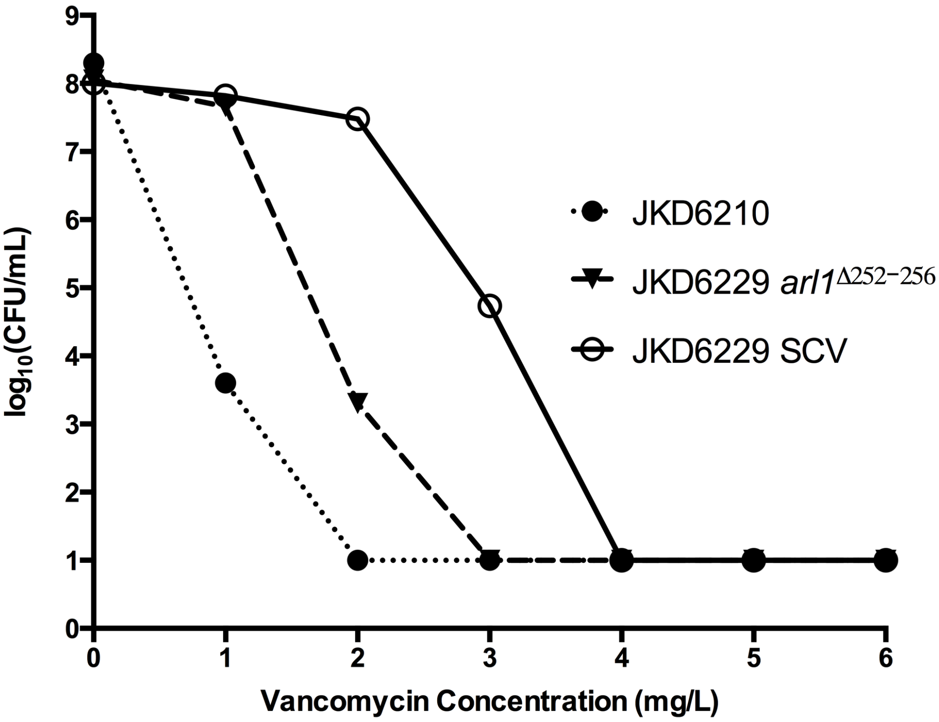

B

Isolate	Vancomycin (mg/L)	Teicoplanin (mg/L)	Oxacillin (mg/L)
JKD6210 (WT)	4	4	12
JKD6229 (SCV)	8	6	>256
JKD6229 (SCV) *alr1* $^{\Delta 256\text{-}256}$	6	4	48

Fig. S2: Functional analysis of *arl1* deletion observed only in Sa_BPH1123. The 5-codon deletion observed in *arl1* (encoding an alanine racemase) was introduced into JKD6229 by allelic exchange and the impact on antibiotic susceptibility was measured by (A) population analysis profile and (B) E-test, showing that the *arl1* mutation in Sa_BPH1123 increased antibiotic susceptibility.

Figure S3

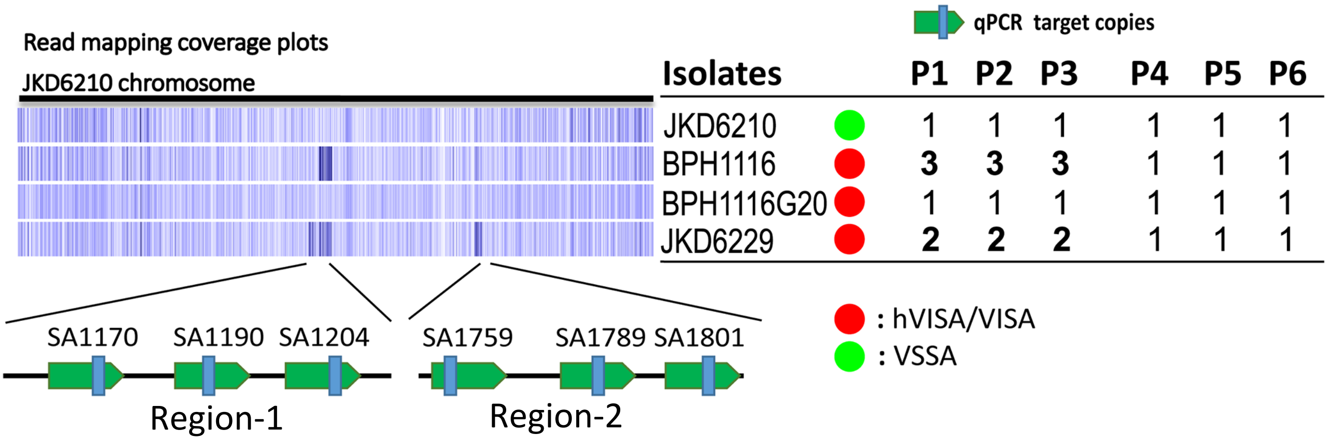

Fig. S3: Assessment of chromosomal expansions on phenotype. *S. aureus* isolate BPH1116 was subjected to 20 serial passages in the absence of antibiotic selection. Heatmap summary of sequence coverage and confirmatory qPCR shows the absence of the 3x 20kb repeats in BPH1116-G20 present in BPH1116. Depicted also are JKD6210 and JKD6229. As for Fig. 4 (main text) P1, P2 and P3 = Region 1 and P4, P5, P6 = Region 2 (phiSA03).
